# The size-distance scaling of real objects and afterimages is equivalent in typical but not reduced visual environments

**DOI:** 10.1007/s00221-025-07064-1

**Published:** 2025-05-12

**Authors:** Amy Siobhan Millard, Irene Sperandio, Philippe A. Chouinard

**Affiliations:** 1 Department of Psychology, Counselling, and Therapy, La Trobe University, Melbourne, Australia; 2 Department of Psychology and Cognitive Science, University of Trento, Trento, Italy

**Keywords:** Size constancy, Afterimages, Emmert’s law, Signal ambiguity theory, Visual environments, Depth cues

## Abstract

Size constancy refers to the human ability to perceive an object as having the same size, despite changes in its retinal image caused by variations in viewing distance. The relationship between perceived size and perceived distance is predicted by Emmert’s law. This study investigated whether the principles of size constancy apply in the same way to afterimages and real objects, hypothesising that perceptual equivalency would result in consistent size-distance scaling constancy for both types of stimuli. Twenty participants completed a size judgment task involving real objects and afterimages presented under binocular, monocular, and complete darkness conditions. Results showed that both types of stimuli adhered to Emmert’s law under binocular conditions; however, afterimages exhibited greater deviations in monocular and dark environments, indicating a breakdown in size constancy. While real objects maintained perceptual scaling even in reduced environments, afterimages displayed diminished accuracy in size and distance perception, especially in darkness. The findings support the signal ambiguity theory, suggesting that afterimages rely more heavily on contextual information due to the lack of stable external references. This study highlights the utility of afterimages as a tool for exploring the limits of visual perception, offering insights into how the visual system handles ambiguous signals.

## Introduction

Size constancy refers to the perceptual ability to maintain a stable perception of an object's size despite variations in its retinal image due to changes in viewing distance (Andrews 1964; Gregory 1973; Morgan 1992). This phenomenon relies on a combination of depth cues and cognitive mechanisms that inform us about our environment (Burton 1945; Howard and Rogers 1995; for a review, see Sperandio and Chouinard 2015). For instance, as we approach a door, its retinal image grows larger, but we perceive its size as constant thanks to depth cues such as perspective, vergence eye movements, and prior knowledge. Despite the robustness of size constancy in everyday vision, the specific cues that enable this mechanism and the way in which these cues interact across different visual environments remain active areas of inquiry.

A key methodological divide in the study of size constancy lies in the type of stimulus used: real objects versus afterimages. Real objects exist in the external world, and their three-dimensional structure allows researchers to examine size constancy in naturalistic settings using objects that are familiar (Bolles and Bailey 1956; Fredebon 1992; Ittelson 1951; Schiffman 1967; Slack 1956) or unfamiliar to an observer (e.g., Hochberg and McAlister 1955; Holway and Boring 1941; Humphrey and Weiskrantz 1969). More recent studies have used digital representations, two-dimensional images of objects displayed on screens (Combe and Wexler 2010; Sperandio et al. 2009), allowing for greater experimental control but potentially introducing differences in how the visual system processes these stimuli compared to three-dimensional objects (Laitin and Witt 2019; Marini et al. 2019; Stefanucci et al. 2015). For the purposes of this study, we categorise both three-dimensional objects and screen-based stimuli as real objects, given their external origin and the fact that they occupy physical space, even when presented digitally.

In contrast, afterimages are internally generated visual experiences resulting from retinal (Brindley 1962; Craik 1940; Virsu 1978; Zaidi et al. 2012) and cortical processes (Shimojo et al. 2001; Sperandio, Lak, et al. 2012a, b; Tsuchiya and Koch 2005). Once induced, they offer a unique advantage for size constancy research: their retinal size remains constant regardless of viewing distance. This feature provides an opportunity to explore size constancy at multiple distances without the need for physical adjustments to the stimulus, which are necessary for real objects. Despite this potential, afterimages have historically been overlooked in the literature, likely due to early studies reporting inconsistent findings that were limited by their induction and measurement methods (Teghtsoonian 1971; Young 1952). However, advancements in technology such as eye-tracking, stimulus presentation software, and digital data collection now enable more precise control and replication of afterimages in studies (e.g., Millard et al. 2020; Sperandio et al. 2013, 2017), making them a promising tool for contemporary research.

A foundational principle for understanding size constancy in afterimages is Emmert's law (Emmert 1881). This law states that the perceived size of an afterimage is proportional to the distance of the surface onto which it is projected. The farther away the surface, the larger the afterimage appears, even though the retinal image size remains constant. This principle exemplifies the role of depth cues in size perception and demonstrates that afterimages, despite being internally generated, can follow the same perceptual rules as external objects when such information is available. Emmert’s law is closely related to the size-distance invariance hypothesis (SDIH), which states that the perceived size of real objects is determined by both retinal image size and perceived distance (Epstein et al. 1961; Howard and Rogers 2002; Kilpatrick and Ittelson 1953).

While afterimages offer certain experimental advantages, they also pose challenges. Unlike real objects, afterimages are subjective experiences that cannot be independently verified by an observer. Furthermore, they are often fleeting phenomena that can be inhibited by the visual system (Daw 1962), raising questions about their relevance in studying perception. However, some evidence suggests that size constancy mechanisms for afterimages and real objects may share similar cognitive processes, such as top-down interpretations of the sensory input (Dwyer et al. 1990; Sperandio et al. 2017) as well as common neural pathways (Kirschfeld 1999; Sperandio, Chouinard et al., 2012a), although direct comparisons between the two remain limited.

Studies in full-cue environments, where binocular vision and good lighting are available, have demonstrated that afterimages can follow the rules of size constancy, similar to real objects (Crookes 1959; Imamura and Nakamizo 2006; Lou 2007). Small deviations from the expected size-distance scaling given by Emmert’s law notably occur for afterimages at greater distances, but the exact point where this happens is debated—whether it is relatively close at approximately 1 m away (Crookes 1959; Lou 2007), or relatively far at over 13 m away (Imamura and Nakamizo 2006). Differences in research goals and experimental design could account for the discrepancy in findings. For instance, closer viewing distances and simple comparison tasks, like those in Crookes (1959) and Lou (2007), may have highlighted small deviations at shorter distances. Even so, in visually restricted environments that involve various degrees of monocular deprivation and low lighting, afterimages appear to be even more prone to misperception than real objects, both in terms of perceived size (Edwards 1953; Hastorf and Kennedy 1957; Irwin 1969) and distance (Furedy and Stanley 1970, 1971). Interestingly, a patient with visual form agnosia was also tested under low lighting and showed a reversal of this typical pattern, where afterimages yielded more accurate size judgements than real objects (Servos 2006). This indicates that some level of size constancy was preserved in their visual system but applied differently to the two stimuli.

Despite the insights provided by these previous studies, inconsistencies in experimental design and a lack of detailed reporting in early work complicate their interpretation. Key factors, such as lighting, the exact methods of visual reduction, and the degree of perceptual equivalence between the stimuli, are not fully documented. While it appears care was often taken to match retinal size or proximal size between real objects and afterimages, most studies fail to account for other important factors, including brightness and colour, which are known to affect the depth perception of a stimulus (Atlı et al. 2020; Pillsbury and Schaefer 1937; Sundet 1978). These elements also influence how similar the two types of stimuli appear and, consequently, how they are processed by the visual system. A more systematic investigation is therefore needed to clarify how these stimuli differ from one another in their ‘size constancy behaviour’ across a range of visual environments.

The present study addresses this gap in the literature by directly comparing size constancy for real objects and afterimages under binocular, monocular, and completely dark conditions. We aimed to determine whether the mechanisms supporting size constancy for real objects and afterimages are the same when both are kept perceptually equivalent, with distinct groups of visual cues available. If they are not, this would suggest that the visual system relies more on certain cues depending on the nature of the stimulus and that the mechanisms of size constancy differ between the two. By directly comparing size constancy for real objects and afterimages, this work bridges distinct areas of research and contributes to a unified understanding of size constancy processes. Testing whether the visual system’s reliance on depth cues is stimulus-dependent provides critical insights into the adaptability and constraints of size-distance scaling mechanisms, revealing how the system meets unique perceptual demands.

Thus, in addition to deviations from Emmert’s law, data on timing and perceived colour were also recorded. This approach allowed us to directly compare other critical components of their appearance and consider their impact on the perceptual reports. To the best of our knowledge, this is the first study to systematically capture detailed participant-reported information on afterimages and real objects under different viewing conditions. We hypothesised that real objects and afterimages, when perceptually matched, would exhibit similar size-distance scaling. Specifically, we expect to observe strong adherence to Emmert’s law in binocular viewing conditions, indicating intact size constancy, moderate deviations under monocular conditions, and significant deviations in complete darkness, which would result from a breakdown in size constancy.

## Methods

### Participants

An a priori power analysis was conducted using G*Power 3.1. Assuming a large effect size (Cohen’s *f* = 0.40; based on our prior work which yielded an average $\eta_{p}^{2}$ = 0.46 and an average Cohen’s *d* = 1.07; Millard et al. 2020), a power of 0.95, and an alpha level of 0.05, the analysis indicated that a sample size of 12 would be sufficient for conducting a two-way ANOVA with three repeated measures. To account for potential variability due to differences introduced by the new experimental conditions, a conservative sample of 20 participants was recruited, along with two additional participants who were recruited separately for pilot testing.

All participants were right-handed and had normal or corrected-to-normal vision. Prior to the experimental session, each participant completed a battery of visual assessments. Eye dominance was determined using the Miles test (Miles 1930). Accommodation and vergence abilities were measured using the Royal Air Force Near Point Rule (Neely 1956). Visual acuity was assessed via a Snellen chart (Snellen 1862), stereoacuity with the Randot Contour Circles Test (Antona et al. 2015), and colour vision using Ishihara’s Test for Colour Deficiency (Ishihara 1918).

The final sample consisted of 20 participants (16 females, 4 males), aged between 19 and 40 years (*M* = 25.85, *SD* = 5.92). Of these, 18 participants were right-eye dominant. All participants met the inclusion criteria for sufficient vergence and accommodation, demonstrating normal accommodative ability for their age range and typical vergence ability, ensuring they could comfortably perform the experimental tasks. No participants exhibited reduced or defective scores that would interfere with their ability to focus on or align with the visual stimuli. There was a minimum visual acuity of 20/30 in the dominant eye, Snellen scores ranged between 20/13 and 20/30. Stereoacuity scores ranged between 20 and 70 s of arc (*M* = 30.75, *SD* = 15.83). No participants were identified as colour blind. Informed consent was obtained from all participants, and ethical approval was granted by the Human Research Ethics Committee of La Trobe University. Participants were compensated for their time with gift vouchers.

### Brief overview

The present study employed a 2 (Stimulus Type: afterimage, real object) × 3 (Visual Environment: binocular, monocular, darkness) repeated-measures design. The stimuli were presented at 10 distances in the visual environments, encompassing a range approximately 2–6 m from the viewer. Each participant completed 60 trials: 30 with afterimages and 30 with real objects, distributed equally across the three visual environments. The experimental setup, stimulus presentation, and task procedures were identical across all trials, with the only variation being the stimulus type.

### Apparatus and stimuli

**Fig. 1:**
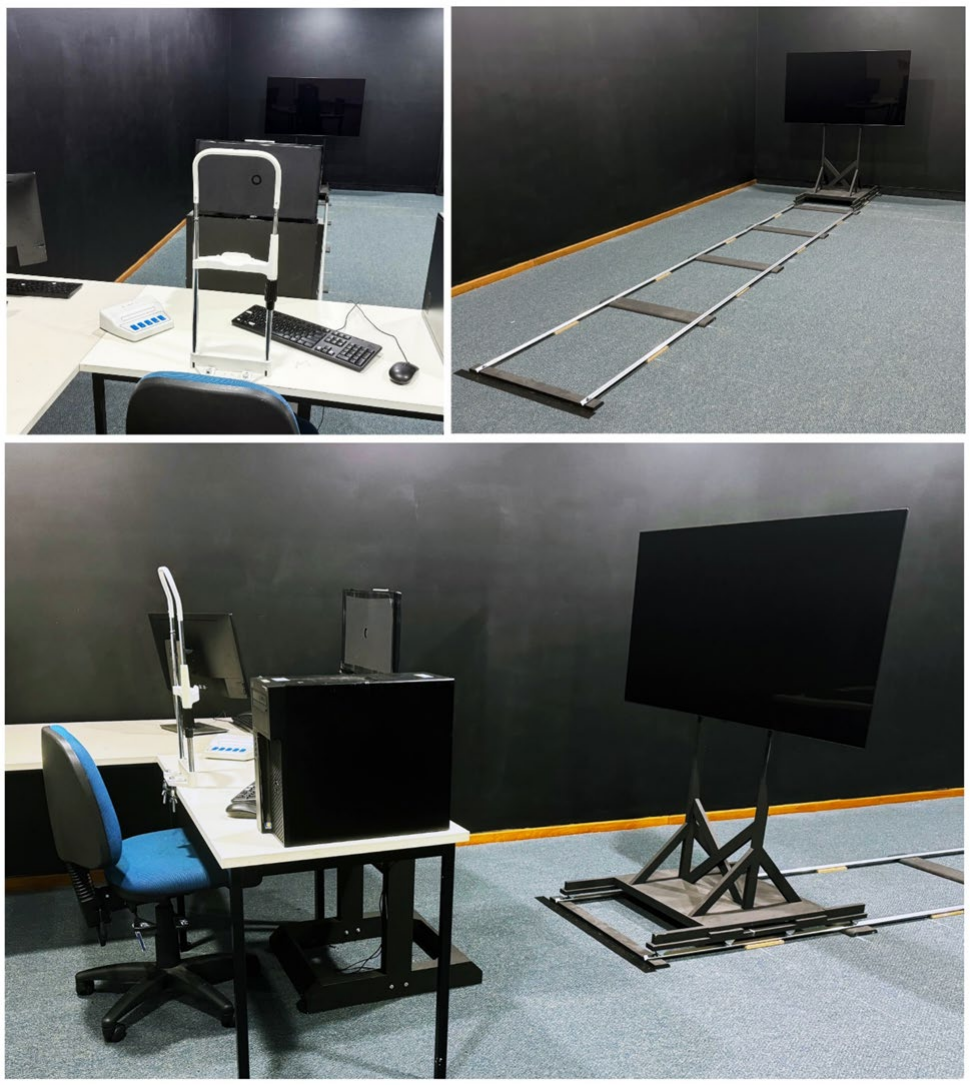
The image shows the apparatus set up in the testing area from different angles (overhead lights are on for visibility, during testing the ambient lighting was dimmer and illuminated by a lamp). The nearby walls were blacked out

**Fig. 2:**
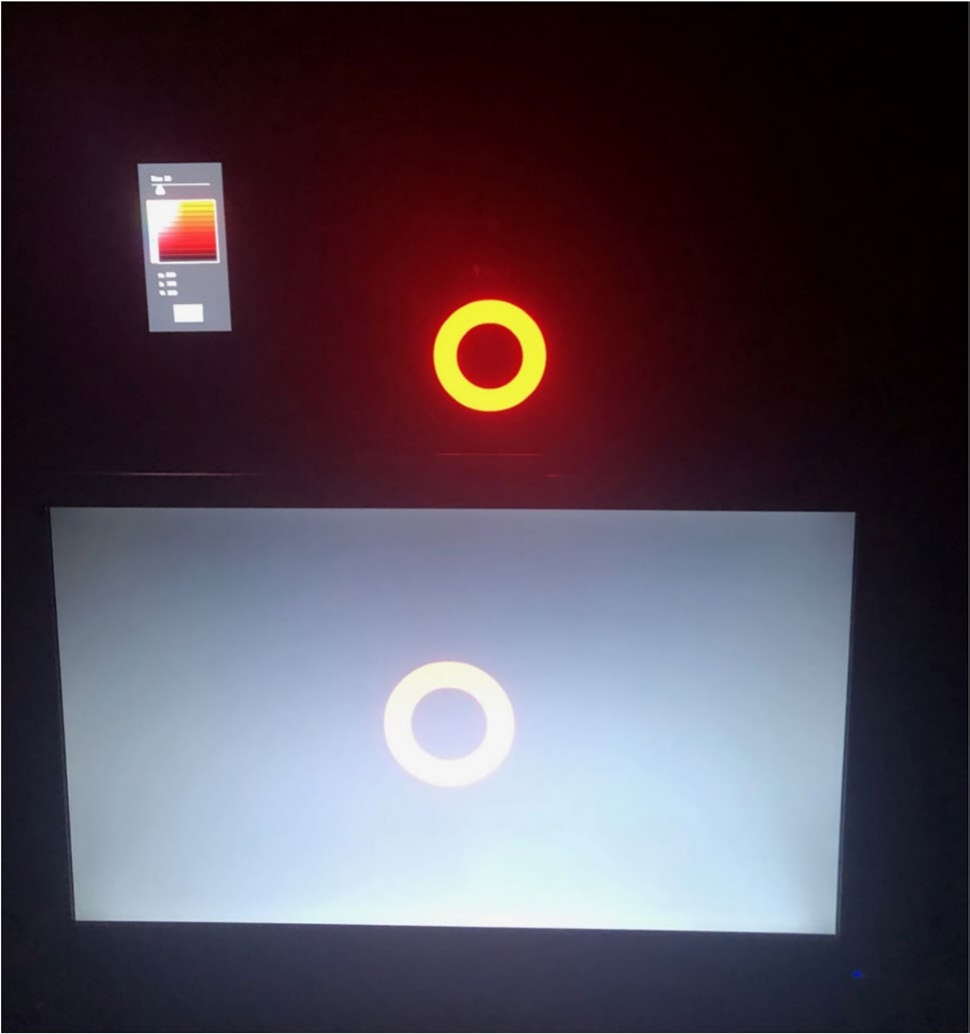
For illustrative purposes, this photograph shows the same red object displayed on a standard screen (front) and on the OLED screen (behind) in the lighting used for the darkness condition. The image demonstrates the residual glow emitted by black pixels on a standard screen compared to the complete lack of luminance from black pixels on the OLED screen, which ensures no ambient light interferes with the perception of stimuli, particularly in the darkness condition

The testing room measured 8 m long by 4 m wide. The experimental apparatus consisted of a testing station equipped with two computers, their peripherals, and a chinrest. Extending back from the centre of the station was an LCD nesting screen (53.52 cm away from the participants’ eyes when situated in the chinrest), and a 55-inch OLED screen mounted on a sliding track (see Fig. 1). The OLED screen was designed to be quickly slid into position at one of 10 distances from the participant, spaced 44 cm apart (the closest distance was 196.20 cm from the participant and the farthest was 590.67 cm). Participants wore noise cancelling headphones playing white noise during testing so that any sounds from re-positioning the OLED would avoid becoming a cue to distance. OLED technology was an optimal choice for this study because of its ability to produce true black levels. Unlike traditional displays, which emit a residual glow even when showing black backgrounds, OLED screens can turn off individual pixels, ensuring that no ambient light interfered with the perception of afterimages or real objects, especially in the darkness condition (see Fig. 2). This feature was critical for maintaining the darkness condition and ensuring high contrast and accurate colour representation for the stimuli and matching tasks displayed on its screen.

The real stimuli consisted of 10 different-sized blue rings that subtended a visual angle of 4.28°. It should be noted that the physical size of the rings was scaled according to distance to maintain a constant retinal image size. Importantly, the appearance of the real stimulus was functionally identical to that of the afterimage. In doing so, both types of stimuli looked the same, regardless of whether they were externally or internally generated. Robust negative afterimages in a shade of blue similar to the real objects were induced by having participants stare at a red ring-shaped inducer (luminance = 7.41 cd/m^2^) for 10 s, after which the afterimages were projected onto the black OLED screen placed at one of 10 possible distances. The calculations for determining size based on visual angle and distance, according to Emmert’s law are as follows:

$$s = d \times \tan \left( \theta \right)$$

where ‘s’ is the perceived size of the object, ‘d’ is the perceived distance of the object, and ‘θ’ is the visual angle subtended by the object (i.e. its retinal image size). According to this law, when the visual angle remains constant, size will change as a function of distance. Correspondingly, apparent distance can also be calculated if size is the known factor.

**Fig. 3:**
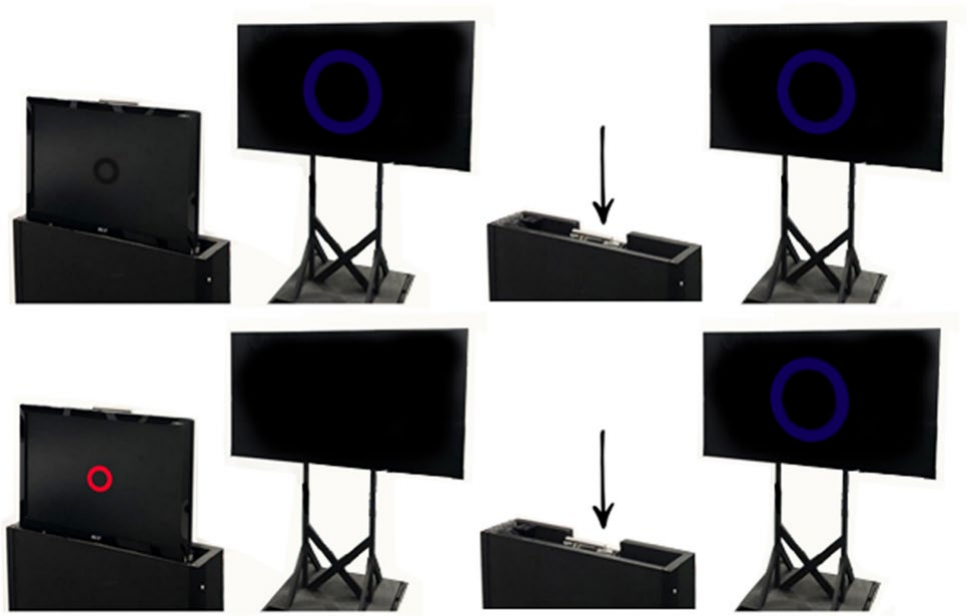
A depiction of the trial sequence for each stimulus type. The top row illustrates the sequence for real-object trials, where the OLED screen revealed a static stimulus after the nesting screen dropped. The bottom row illustrates the sequence for afterimage trials, where the LCD nesting screen induced an afterimage before dropping to reveal the black OLED screen. The nesting screen was entirely blacked out, except for a central cut-out (4 cm ring), which provided a consistent fixation point during the start of each trial

The LCD nesting screen served as both inducer and blocking screen. When participants were seated with their head stabilized on a chinrest, the raised nesting screen completely obscured the OLED screen from view. A lever mechanism allowed it to swiftly drop out of view into the nesting box to reveal the stimulus on the OLED screen in each trial. The nesting screen was completely blacked out with material, except for a 4 cm ring shaped cut-out in the centre (also subtending 4.28° of visual angle), which provided a consistent point of central fixation at the start of each trial. In afterimage trials, the screen under the material was turned on, displaying a glowing red inducing ring. In real object trials, this screen was turned off, displaying a dark black which cast no light (see Fig. 3).

### Procedure

#### General procedure

Each trial consisted of three phases: (1) fixation, (2) observation, and (3) matching. For each trial, participants were presented with a stimulus (either a real object or an afterimage) at one of ten distances. These trials were conducted under binocular, monocular, or dark visual environments, counterbalanced across participants using a Latin square design. The order of distances was randomised within each environment. Some elements of this procedure are required for working with afterimages (e.g., induction), so a modified version of this order of operations was kept for real objects to ensure the experience was as identical as possible.

During the fixation phase (1), participants fixated on a ring displayed on a nesting screen for 10 s. In the afterimage trials, where the ring glowed red, this fixation induced afterimages. In the real object trials, the ring did not emit light and therefore did not induce afterimages, however, the fixation period was kept consistent. The nesting screen was subsequently lowered using a lever mechanism, allowing it to drop instantly like a guillotine into the nesting box and out of view, revealing the OLED display with either the blank screen (for afterimage trials) or a corresponding image of a ring on screen (for real object trials).

E-Prime 2.0 Professional software (Psychology Software Tools, Inc.) was used to control the timing and presentation of the stimuli, with a Chronos Response pad attached for participant use. During the observation phase (2), participants were instructed to indicate with a Chronos button press when they began to see the stimulus on the OLED screen (i.e., an afterimage or a physical object), verbally report a magnitude estimate of its perceived distance, and then press the button again to signal the end of their observations. For afterimages, this was when the stimulus had faded from view; for real objects, which do not dissipate on their own, this was when participants felt they could recreate what they had observed.

The matching phase (3) occurred immediately after observation was indicated to be over. A matching tool was presented on the OLED screen directly after the second button press by switching to its full-screen application with a keyboard shortcut. In each trial, to prevent participants from relying on retinal size as a reference point the matching tool would show a ring of randomised size, ranging between 1 and 67 cm. This ensured that adjustments reflected perceived judgments informed by distance and depth cues. The colour was also randomised, ranging between almost the full spectrum of hues, saturations, and values available using a HSV colour model (values of less than 5% were not used to ensure it was easily visible on screen). Participants were able to freely adjust these elements until the ring matched their perception of the stimulus they had just seen. Once participants were satisfied with their match the data was saved, completing the trial sequence.

#### Matching tool

**Fig. 4:**
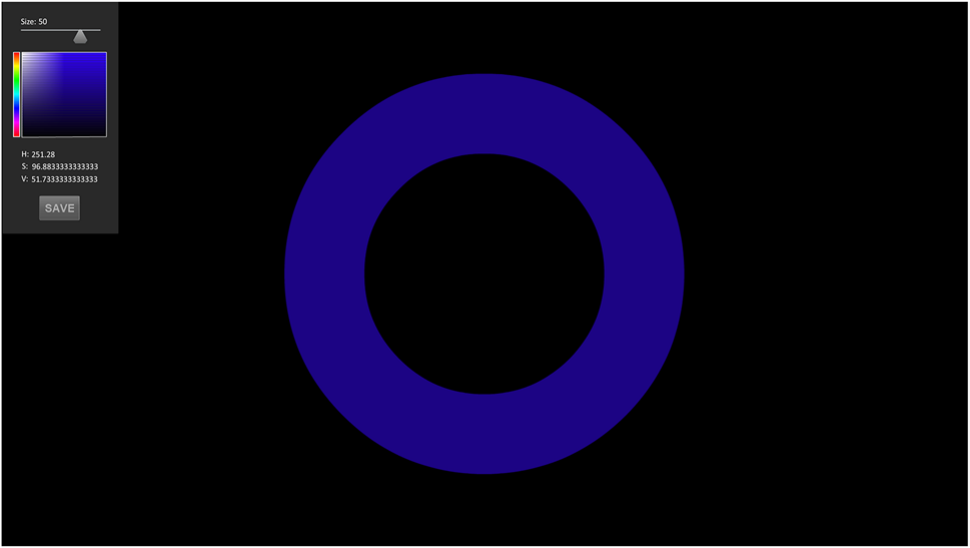
Sample screenshot from the matching task participants completed at the end of every trial. Although it recorded technical information (exact diameter, hue, saturation, and value), the tool was designed for quick and intuitive use and required no knowledge of these factors. Participants simply used keystrokes or mouse clicks to adjust the randomised ring until they felt it resembled what they had just seen. Participants were familiarised with how to quickly navigate the tool during practice trials, allowing for most matches to be completed in seconds

Adobe Animate CC software (Adobe Systems Incorporated, San Jose, CA, USA) was used to create the matching tool, which consisted of an adjustable ring with a size bar and colour picker box (see Fig. 4). Modifications to size and colour could be rapidly made using keyboard strokes or mouse clicks. This approach allowed us to comprehensively capture participants' perceptual experiences as quickly as possible after a stimulus was no longer present in the visual field. Though some reliance on memory is necessary, paradigms that involve real time simultaneous presentation of the comparison and the target stimuli cannot be applied to afterimages. Since afterimages are attached to the retina, they would move with one’s gaze away from the projection surface and overlay the comparison stimulus. However, with this novel tool, participants had a fast and intuitive way to visually report what they had just experienced in terms of size and colour, while the generated output provided the nuanced details of ring diameter (centimetres), and multiple dimensions of colour (hue, saturation, and value).

#### Visual environments

Three visual environments were tested: binocular, monocular, and complete darkness. The binocular condition provided participants with the full array of depth cues, including stereopsis, vergence, and monocular cues (e.g., accommodation, perspective, texture gradient), representing typical daily viewing. Monocular viewing reduced depth information by removing binocular cues, simulating situations where one eye is obstructed or binocular depth information is limited. The dark condition nearly eliminated all pictorial depth cues, leaving only the retinal image size of the stimulus and oculomotor adjustments (such as tonic accommodation and vergence, which refers to the default eye alignment at rest). While stereopsis theoretically remains possible in this condition, its effectiveness as a cue in the dark is significantly diminished without surrounding visual references. This set-up mimicked environments with severely restricted visual input, such as night vision.

The visual environments were created by using PLATO Visual Occlusion Spectacles, controlled by E-Prime combined with adjustments to ambient lighting. In the binocular condition, both lenses remained transparent, and diffuse room lighting from a lamp enabled full use of depth cues and visibility of afterimages in the testing area while keeping the peripheral spaces dim (average luminance = 100 cd/m^2^, for context, bright overhead room lighting would yield a luminance of approximately 400 cd/m^2^). In the monocular condition, the non-dominant eye was occluded just before the nesting screen dropped, which limited binocular cues. This was done to reduce variability in visual performance that might arise from occluding the dominant eye, which could impair overall vision more severely. In the darkness condition, both lenses were transparent like in binocular conditions, but room lighting was extinguished (average luminance = .003 cd/m^2^) removing all pictorial cues. Despite the very dark surroundings, having the spectacles transparent ensured that no additional visual obstructions or distortions were introduced by the lenses themselves, isolating the effect of the dark environment.

#### Pilot testing

Before beginning the experiment, we conducted pilot testing to ensure that afterimages and real objects were as perceptually equivalent as possible. One session determined the hue and luminance for the real objects based on what could be reliably produced among different individuals from afterimage inducers of various colours in the testing space. A bright red inducer which produced a distinct blue-purple afterimage on the black OLED screen was chosen. Sessions of full experimental run-throughs were then conducted with two pilot participants (their data is not included in the final sample) to fine-tune the dark visual environment and control for any elements in the room that unexpectedly reflected or cast light.

### Measures

From these procedures, we obtained seven dependant variables of interest: i) Size constancy measures: perceived size and perceived distance; ii) timing measures: point of awareness and observation time; and iii) colour measures: hue, saturation, and value.

Judgments of size and distance were assessed for their adherence to Emmert's law. In the case of size, this measure captures how much perceived size deviated from the theoretically perfectly size-distance scaled appearances of the stimuli, as calculated using Emmert’s law, for each distance tested. For distance, this measure captures how much perceived distance deviated from the actual distances used in the calculations of Emmert’s law to predict size. We collectively use the term ‘deviations from Emmert’s law’ to refer to both measures, although classically Emmert’s law is used to explain changes in size, not distance. Raw ratings of size were collected from the matching tool, while raw ratings of distance were given verbally as magnitude estimations (which were first standardised into z-scores).

To calculate deviations from Emmert’s law, we used a three-step regression analysis following the methodology outlined in Millard et al. (2020). First, a linear regression was fitted to participants’ size estimates across distances to calculate the observed slope. Second, a separate regression modelled the theoretical slope predicted by Emmert’s law, where perceived size scales proportionally to retinal size and perceived distance. Finally, the absolute difference between the observed and theoretical slopes was calculated to quantify deviations from size-distance scaling. These deviations reflect participants’ ability to integrate retinal size and perceived distance into size judgments, a hallmark of size constancy. Further details on this calculation can be found in Millard et al. (2020). For distance, the predictions made by Emmert’s law were also standardised into z-scores before the regression slope calculations were performed. Finally, for ease of communication the indices were converted into percentage representing how much they deviated from Emmert’s law (deviation/slope of Emmert’s law*100), where 0% indicates no deviation and, therefore, perfect size constancy, while 100% indicates complete deviation and a breakdown in size constancy.

For timing measures, the point of awareness was measured as the interval of time in milliseconds between the removal of the nesting screen and the participant’s first button press on the response pad to indicate that the stimulus was seen. The observation time was measured as the interval of time in milliseconds between the first and the second button press. These values were then converted to seconds for ease of understanding. Such timing measures are traditionally denoted in afterimage research as ‘onset’ and ‘duration’; however, we required a version that is applicable to the procedures for both stimuli.

Colour measures of hue, saturation, and value were recorded from the outputs of the matching tool. The HSV colour space is a geometric representation of these three dimensions of colour; each represents an axis in the colour space that defines the properties of a given colour. Hue is the type of colour and was measured as an angle on the colour wheel, ranging from 0° to 360° (with red at 0°, green at 120°, blue at 240°, and so on around the wheel). Saturation is the intensity or purity of the colour and was measured as a percentage from 0 to 100%. At 100%, the colour is fully saturated, while at 0%, the colour is completely desaturated, appearing as a shade of grey. Value is the brightness or luminance of the colour and was also measured as a percentage from 0 to 100%. At 100%, the colour is at its brightest, and at 0%, the colour is completely black. In short, hue forms the base colour, saturation indicates the intensity, and value indicates the brightness.

### Data analysis

An analysis of outliers was conducted across all measures to ensure reliability and accuracy of participant responses. The dataset comprised seven measures (size, distance, point of awareness, observation time, hue, saturation, and value), two types of stimulus (real and afterimage), and three types of visual environments (binocular, monocular, and darkness). Each participant completed trials at ten different distances for each combination of object type and visual environment, resulting in 60 trials per condition for a total of 1200 trials per measure. Consequently, we collected 8400 data points (i.e., 1200 trials × 7 measures). Outlier detection was performed by examining the distribution of data points within each measure (1200 points per measure). Values that deviated more than three standard deviations from the mean were considered as outliers and excluded from the analyses. This criterion led to the removal of 80 data points in total, representing less than 1% of the entire dataset.

Data were analysed using JASP Version 0.18.3 (JASP Team 2024) and GraphPad Prism version 6 (GraphPad Software, Inc.; La Jolla, CA, United States). A two-way repeated measures ANOVA with Stimulus type (real vs. afterimage) and Visual environment (binocular vs. monocular vs. darkness) was conducted on each dependant variable. Greenhouse–Geisser corrections were applied where necessary. Post-hoc pairwise comparisons were performed using Holm corrections to account for multiple comparisons. Effect sizes are reported as partial eta-squared (*η*_*p*_^*2*^) or ANOVA results and Cohen’s *d* for pairwise comparisons. Bayesian ANOVAs were also conducted to leverage the established decision-making framework of NHST, while also utilising the Bayesian approach to provide a richer, probabilistic interpretation of the evidence. Pearson’s correlation coefficients (*r*) were calculated to assess the relationships between size-distance scaling and visual acuity, stereoacuity, observation time, and value. Observation time and value were included as indicators of afterimage strength. Effect sizes for the correlations were interpreted using Cohen’s guidelines, where* r* values of .10, .30, and .50 represent small, medium, and large effects, respectively. In addition to reporting Pearson correlation coefficients, we calculated the coefficient of determination (*R*^2^) by squaring the *r* values. This represents the proportion of variance in one variable that can be explained by the variance in the other as a percentage.

Further, using the same procedures as outlined in our previous work (see Millard et al. 2020), we performed two supplementary analyses to examine the more fine-grained differences in size-distance scaling across the different visual environments. The regression slopes for size and distance judgements in each testing condition were normalised and then subtracted from each other in order of most to least visual information (binocular—monocular, then monocular—darkness) to determine the relative contribution of the groups of depth cues available in those environments. In other words, this analysis allows us to quantify the weight of each group of depth cues in the perceptual scaling of size and distance, showing how each set of cues influences judgments relative to one another across different visual environments. Note that these values do not add up to 100% due to rounding. To determine at which distance the contributions of different groups of cues did not overlap (i.e., uniquely contributed), the regression slopes were also plotted to evaluate the mean perceived size with 95% confidence intervals as a continuous function of distance for each environment.

Finally, to provide additional context for interpreting our findings, we employed a benchmarking model based on results from previous comparable studies. For real objects, we derived benchmarks from Holway and Boring’s classic (1941) study, which systematically measured deviations from Emmert’s law under binocular, monocular, and darkness conditions (among others). These values were converted to percentages to align with our study’s deviation metric. Specifically, benchmarks were set at 7% for binocular, 19% for monocular, and 92% for darkness, reflecting their findings under similar experimental conditions. For afterimages, we used the deviation patterns reported in Millard et al. (2020), which was designed as a comparable afterimage paradigm of Holway and Boring’s original work, to establish benchmarks. Those results were also converted to percentages, and were set at 20% for binocular, 47% for monocular, and 100% for darkness. While these benchmarks offer valuable context, key methodological differences between studies, such as additional cue reduction under darkness in the original works and differing distance ranges were considered when interpreting the comparisons.

## Results

### Overall summary

#### Size constancy

Real objects adhered to Emmert’s law more consistently than afterimages in the reduced visual environments. Under binocular conditions, both real objects and afterimages demonstrated accurate size constancy. However, in monocular and dark environments, afterimages deviated more significantly from Emmert’s law, showing reduced size-distance scaling. While real objects maintained consistent size perception in monocular conditions, afterimages exhibited larger deviations in size perception, particularly in darkness, where size underestimation was observed for both types of stimuli. Distance estimates for real objects and afterimages remained aligned with Emmert’s law under binocular viewing, but deviations emerged in monocular and dark environments. Perceptual judgments of distance were less accurate for afterimages than for real objects, particularly in darkness, where both types of stimuli were misperceived as closer than they actually were. The interaction between visual environment and stimulus type was driven by these discrepancies in perceived size and distance.

#### Timing

Unsurprisingly, participants became aware of real objects faster than afterimages across all visual environments, with the largest delays for both types of stimuli in dark conditions. No significant timing differences were found for real objects across viewing conditions. In contrast, afterimages took significantly longer to reach awareness under dark conditions.

#### Colour

There were no significant differences in hue or saturation between real objects and afterimages across different visual environments, although real objects were consistently perceived as brighter than afterimages. Participant judgements of both stimuli were close to, but not fully aligned with the physical features of the real objects.

### Size constancy

#### Size (% of deviation from Emmert’s law)

**Fig. 5:**
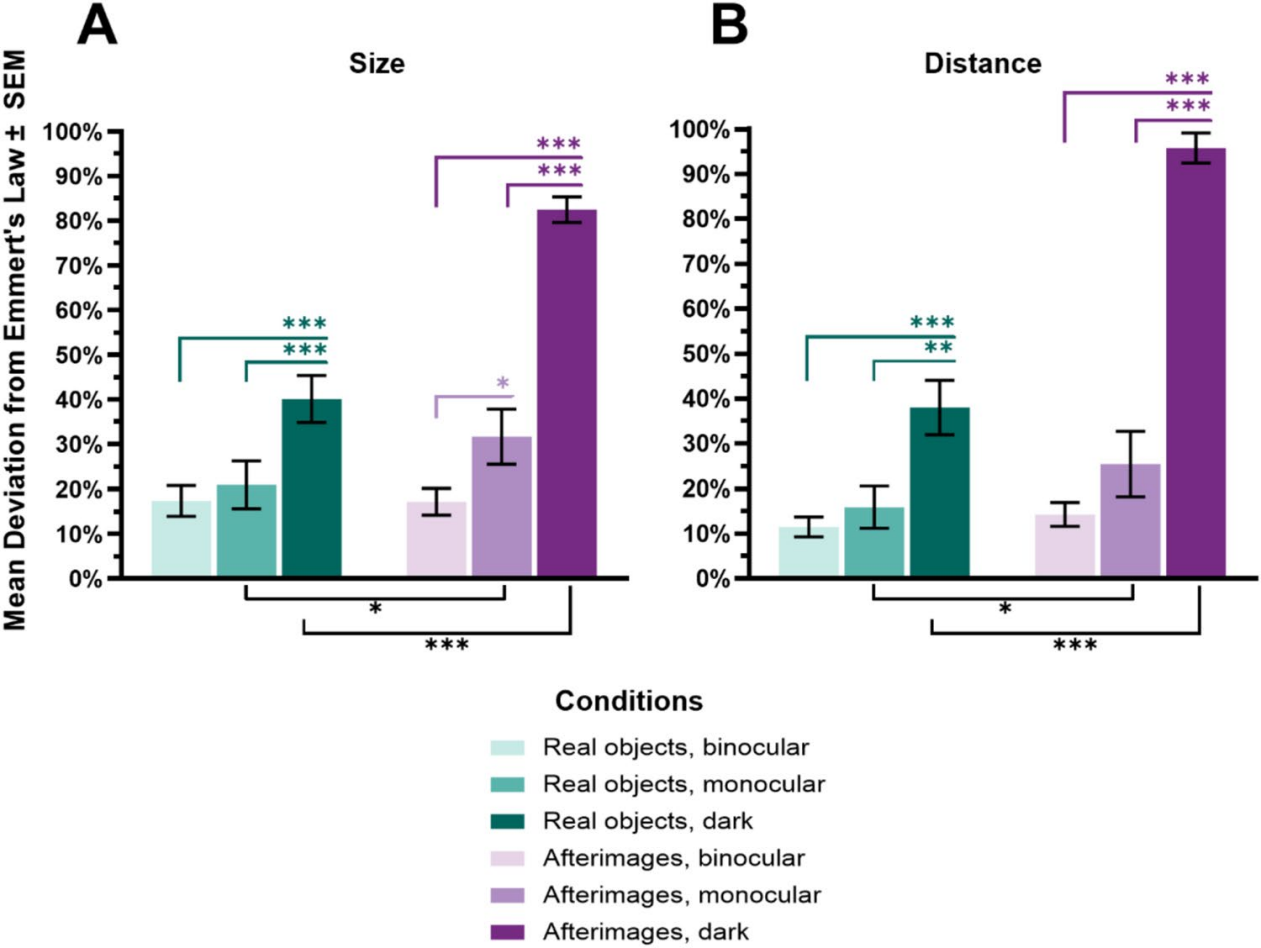
Means and standard errors (SEM) for Size (**A**) and Distance (**B**; deviation from the theoretical slope calculated from Emmert’s law; the x-axis corresponds to the matching testing conditions in the legend in all graphs). Significance indicators above the x-axis show differences between environments for the stimuli types, significance indicators below the x-axis show differences between stimuli types across environments. *p < .05, **p < .01, ***p < .001. The bars are colour coded based on condition: teal for real objects, purple for afterimages, lightest colour for binocular, medium colour for monocular, darkest colour for darkness conditions

The ANOVA demonstrated a main effect of Visual environment ($F_{{\left( {2,38} \right)}}$ = 79.40, *p* < .001, *η*_*p*_^*2*^ = .81, *BF*_10_ > 1000), a main effect of Stimulus type ($F_{{\left( {1,19} \right)}}$ = 31.37, *p* < .001, $\eta_{p}^{2}$ = .62, *BF*_10_ = 80.53), and an interaction between the two factors ($F_{{\left( {2,38} \right)}}$ = 25.73, *p* < .001, *η*_*p*_^*2*^ = .58, *BF*_10_ > 1000; see Fig. 5).

Pairwise comparisons (see Tables 1 and 2) revealed that in the binocular environment, judgements of size were accurate and scaled with the predictions made by Emmert’s law for both real objects and afterimages. When making judgements about the size of real objects, perceptual reports were unaffected by viewing conditions. In contrast, size estimates of afterimages were generally less accurate than those of real objects. Moreover, afterimages under restricted viewing conditions were judged less accurately than under full viewing conditions. Finally, the interaction was primarily driven by the perception of afterimages in the environment, where their perceived size deviated more from Emmert’s law than that of real objects, as well as afterimages under both binocular and monocular conditions.

**Table 1:** Means, standard deviations, and ranges for size and distance (deviation from the theoretical slope calculated from Emmert’s law) in all conditions

Condition		*M*	*SD*	Range
*Size*				
Real objects	Binocular	17.35	15.25	.45–50.05
	Monocular	20.95	23.7	.64–104.80
	Dark	40.12	23.46	7.32–88.17
Afterimages	Binocular	17.19	13.31	.10–53.01
	Monocular	31.68	27.57	1.74–117.80
	Dark	82.46	12.86	51.53–100.55
*Distance*				
Real objects	Binocular	11.5	9.87	1.19–48.72
	Monocular	15.87	20.89	2.18–142.49
	Dark	38.01	27.08	55.53–122.52
Afterimages	Binocular	14.27	11.91	1.41–33.34
	Monocular	25.44	32.54	1.19–98.53
	Dark	95.79	14.92	3.63–107.67

**Table 2:** Post hoc tests for size and distance (deviation from the theoretical slope calculated from Emmert’s law) showing t-statistics, *p* values, Cohen’s d, and Bayesian factors

Comparison				*t*	*Pholm*	*d*	*BF10*
*Size*							
Binocular	Real objects	–	Afterimages	.04	.97	.01	.23
Monocular				− 2.52	.02*	− .53	2.79
Dark				− 6.99	< .001***	− 2.1	> 1000
Real objects	Binocular	–	Monocular	− .82	.43	− .18	.31
	Binocular	–	Dark	− 4.27	.001**	− 1.13	72.22
	Monocular	–	Dark	− 4.31	.001**	− 0.95	85.34
Afterimages	Binocular	–	Monocular	− 2.7	.01*	− .72	3.82
	Binocular	–	Dark	− 18.02	< .001***	− 3.23	> 1000
	Monocular	–	Dark	− 9.46	< .001***	− 2.51	> 1000
*Distance*							
Binocular	Real objects	–	Afterimages	− 1.15	.27	− .13	.41
Monocular				− 2.23	.04*	− .45	1.74
Dark				− 10.9	< .001***	− 2.73	> 1000
Real objects	Binocular	–	Monocular	− 1.06	.30	− .21	.38
	Binocular	–	Dark	− 4.56	< .001***	− 1.25	> 100
	Monocular	–	Dark	− 3.45	.002**	− 1.05	15.64
Afterimages	Binocular	–	Monocular	− 1.8	.09	− .53	.91
	Binocular	–	Dark	− 20.18	< .001***	− 3.85	> 1000
	Monocular	–	Dark	− 9.98	< .001***	− 3.32	> 1000

*p < .05, **p < .01, ***p < .001

#### Distance (% of deviation from Emmert’s law)

The ANOVA demonstrated a main effect of Visual environment (*F*_(2, 38)_ = 73.60, *p* < .001, *η*_*p*_^*2*^ = .80, *BF*_10_ > 1000), a main effect of Stimulus type (*F*_(1, 19)_ = 115.85, *p* < .001, *η*_*p*_^*2*^ = .86, *BF*_10_ > 100), and an interaction between the two factors (*F*_(2, 38)_ = 47.17, *p* < .001, *η*_*p*_^*2*^ = .71, *BF*_10_ > 1000; see Fig. 5).

Pairwise comparisons (see Tables 1 and 2) revealed that ratings of distance did not strongly deviate from Emmert’s law for real objects or afterimages in the binocular environment. In the monocular condition, while there was no significant difference between the monocular and binocular ratings for either stimulus type, the perceived distance accuracy was lower for afterimages than for real objects. The interaction effect was primarily driven by the dark condition. Here, the perceived distance for both real objects and afterimages was less accurate compared to both binocular and monocular conditions, with afterimages showing greater inaccuracy than real objects.

#### Correlations between size and distance (% of deviation from Emmert’s law)

As depicted in Table 3, real objects showed strong proportional relationships between size and distance in monocular and dark conditions, suggesting consistent perceptual scaling under these reduced cue circumstances. This consistency between size and distance was not observed under binocular viewing where there was the greatest adherence to Emmert’s law. In contrast, afterimages displayed a different pattern of correlations, with significant moderate relationships between size and distance under both binocular and monocular conditions, but not in darkness. This suggests that while size and distance ratings of afterimages deviate greatly from Emmert’s law in the dark, these misperceptions appear to occur independently.

**Table 3:** Correlational relationships of size and distance between the variables, including r coefficients, *p* values, Cohen’s effect sizes, and R^2^

Pearson correlation	*r*	*p*	Effect size	R^2^
*Between size and distance, for real objects*				
Binocularly	.25	.28	Small	.06
Monocularly	.73	< .001***	Large	.54
In darkness	.70	< .001***	Large	.49
*Between size and distance, for afterimages*				
Binocularly	.55	.01*	Medium	.30
Monocularly	.54	.01*	Medium	.29
In darkness	− .08	.75	Small	.01
*For size, between object types*				
Binocularly	.38	.10	Medium	.14
Monocularly	.73	< .001***	Large	.54
In darkness	− .03	.90	Small	.00
*For distance, between object types*				
Binocularly	.52	.02*	Large	.27
Monocularly	.83	< .001***	Large	.69
In darkness	.49	.03*	Medium	.24

*p < .05, **p < .01, ***p < .001

Further, misperceptions of distance appeared to be more consistent across stimulus types than misperceptions of size. Significant relationships were observed in all the tested environments, indicating a stable relationship between how distance was misperceived for both real objects and afterimages. Conversely, misperceptions of size were more variable; a significant relationship between the misjudgements of afterimages and real objects was only observed under monocular conditions.

Across all stimuli-environment conditions, the relationships between visual acuity, stereoacuity, observation time, and value with deviations from Emmert’s law were weak and non-significant. Visual acuity and stereoacuity explained very little variance in size and distance misperceptions (*r* ranged from − .28 to .24,* p* = .22 to .98, *R*^*2*^ = .00 to .08), indicating that individual differences in visual abilities did not systematically impact size-distance scaling. Similarly, observation time was not significantly correlated with deviations from Emmert’s law for either size or distance judgments (*r* = − .21 to .34, *p* = .14 to .95, *R*^*2*^ = .04 to .12), suggesting that shorter afterimage persistence (i.e., duration in the visual field) did not negatively affect size-distance scaling, and for real objects, shorter observation times did not impair judgment accuracy. Perceived value also showed no significant relationship with size or distance judgments (*r* = − .41 to .40, *p* = .07 to .90, *R*^*2*^ = .16 to .17), indicating that afterimage brightness or intensity did not influence scaling abilities, nor did the perceived visibility of real objects. Overall, none of these factors explained substantial variance in size and distance misperceptions, suggesting that differences in visual abilities, viewing duration, and brightness had minimal impact on deviations from Emmert’s law in this dataset.

#### Relative impact of depth cues (mean with 95% CI)

**Fig. 6:**
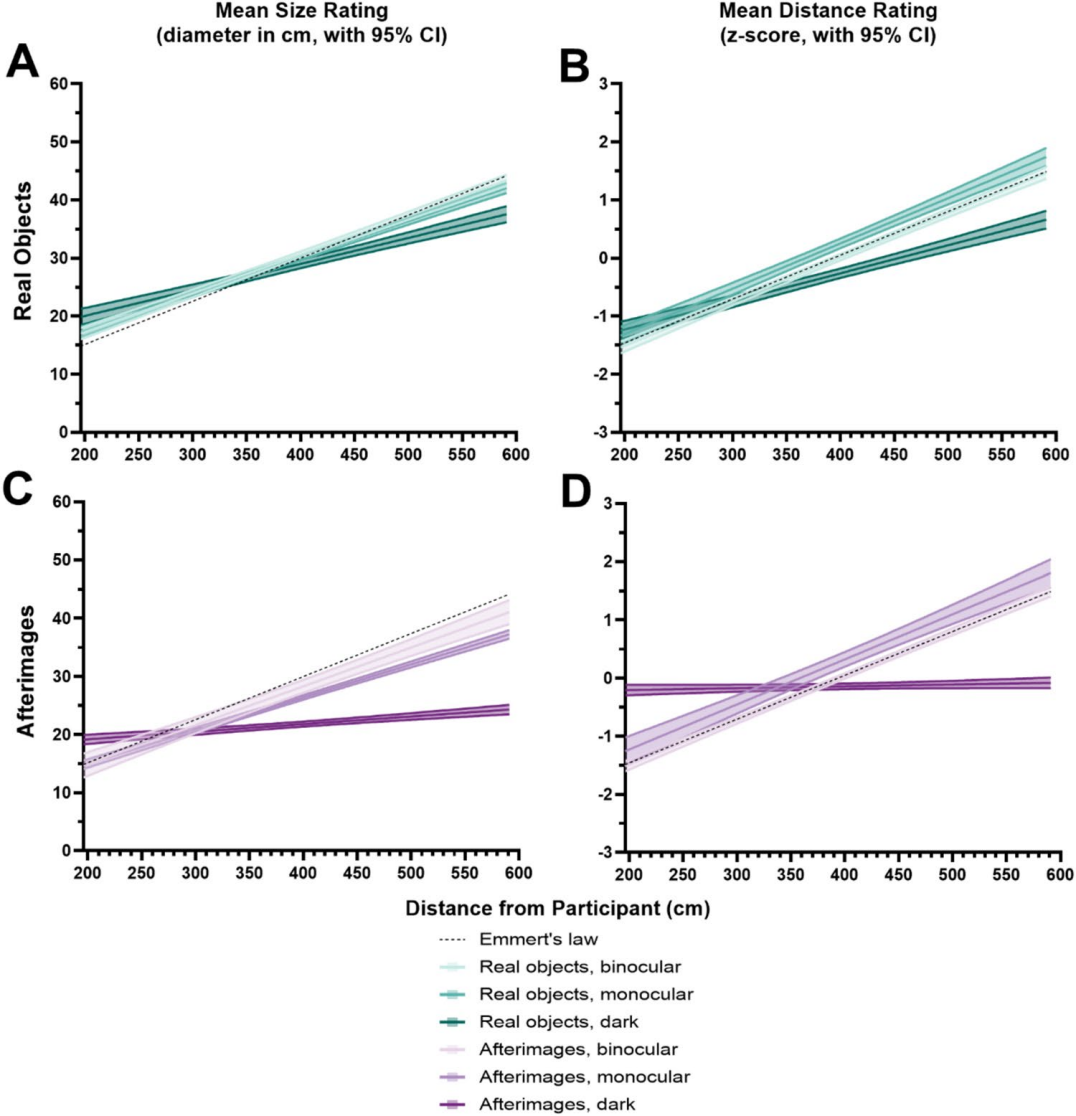
Mean and 95% confidence intervals for size (diameter of ring in cm) and distance judgements (magnitude estimations converted to z-scores) made in the three different visual environments from all participants, plotted as a function of distance. The plots are colour coded based on condition: **A** and **B** are teal for real objects, **C** and **D** are purple for afterimages, lightest colour for binocular, medium colour for monocular, darkest colour for darkness. The dashed line represents the theoretically perfect slope of Emmert’s law and is plotted identically across all four graphs as a frame of reference. In **A**, the close overlap of the binocular and monocular condition lines reflects the similarity in participants’ size judgments under these conditions across most distances. This overlap aligns with the reported results, which show no significant enhancement of precision in binocular conditions compared to monocular ones. Divergences in the darkness condition are more apparent, consistent with theoretical predictions about reduced depth cue availability in that environment

Based on the plotted regression slopes for *size judgments of real objects* (see Fig. 6A), binocular and monocular conditions were similar across all distances, while the darkness condition diverged at distances beyond 415 cm (distance 6). Binocular cues did not enhance precision compared to monocular cues, even though both conditions aligned more with Emmert’s law at greater distances. Closer distances showed slight over-constancy, where objects appeared larger than they actually were. In darkness, objects appeared smaller at farther distances. Binocular cues contributed 3.19% to size scaling, monocular cues 16.31%, and the residual cues in darkness 74.91%.

Based on the plotted regression slopes for *distance judgments of real objects* (see Fig. 6B)*,* the binocular condition closely followed Emmert’s law. Conversely, monocular judgments of distance diverged from Emmert’s law beyond 283 cm (distance 3) of viewing distance, with objects misperceived as farther away than they actually were, and in darkness, objects were seen as closer beyond 371 cm (distance 5). At closer distances, objects appeared farther away in darkness. Binocular cues improved the precision of distance judgments, contributing 1.17% to distance scaling, while monocular cues contributed 20.28%, and the residual cues in darkness accounted for 78.11%.

Based on the plotted regression slopes, *size judgments of afterimages* (see Fig. 6C) adhered to Emmert’s law up to 371 cm (distance 5), beyond which under-constancy was observed, with afterimages appearing smaller than their predicted size. Monocular judgments diverged from binocular judgements at this same distance point, with more pronounced shrinking at farther distances. In darkness, afterimages showed a flat slope from 240 cm (distance 2) to 327 cm (distance 4), with a consistent perception of approximately 18 cm-sized rings across all distances. Binocular cues contributed 8.08% to size-distance scaling, monocular cues 56.55%, and darkness 29.84%.

Based on the plotted regression slopes for *distance judgments of afterimages* (see Fig. 6D), binocular conditions entirely aligned with Emmert’s law, while monocular judgments consistently overestimated distances beyond 283 cm (distance 3). In darkness, ratings again showed a flat slope intersecting with the other conditions at midrange, indicating an intermediate perceived distance across all distances. Binocular cues contributed .86% to distance scaling, monocular cues 90.59%, and darkness 8.03%.

#### Benchmarking model (one-sample t-tests)

We conducted one-sample t-tests to compare our observed deviations for real objects against the benchmarks for each condition derived from Holway and Boring’s (1941) work. Observed deviations in our binocular condition (*M* = *17.35%, SD* = *15.25%*) were greater than the benchmark (7%), indicating less adherence to Emmert’s law: *t*(19) = 3.04, *p* = .007, *d* = .68. However, monocular deviations (*M* = 20.95%,* SD* = 23.70%) were aligned with the benchmark (19%): *t*(19) = .37, *p* = .72, *d* = .08. In darkness, our observed deviations (*M* = 40.12%,* SD* = 23.46%) were smaller than the benchmark (92%), indicating greater adherence to Emmert’s law: *t*(19) = − 9.89, *p* < .001, *d* = − 2.21.

Further one-sample t-tests were used to compare our observed deviations for afterimages against the benchmarks for each condition derived from our previous afterimage work (Millard et al. 2020). Observed deviations (*M* = 17.19%, *SD* = 13.32%) were not significantly different from the benchmark (20%), indicating consistency in our findings for afterimages under binocular conditions: *t*(19) = − .82, *p* = .36, *d* = − .18. Monocularly, deviations were significantly smaller (*M* = 31.69%, *SD* = 27.57%) than the benchmark (47%), suggesting better size constancy for afterimages in our monocular condition compared to previous findings: *t*(19) = − 2.42, *p* = .03, *d* = − .54. Similarly, our observed deviations in darkness (*M* = 82.46%, *SD* = 12.86%) were again smaller than the benchmark (100%), indicating greater adherence to Emmert’s law in the present study: *t*(19) = − 6.03, *p* < .001, *d* = − 1.35.

### Timing

#### Point of awareness (s)

**Fig. 7:**
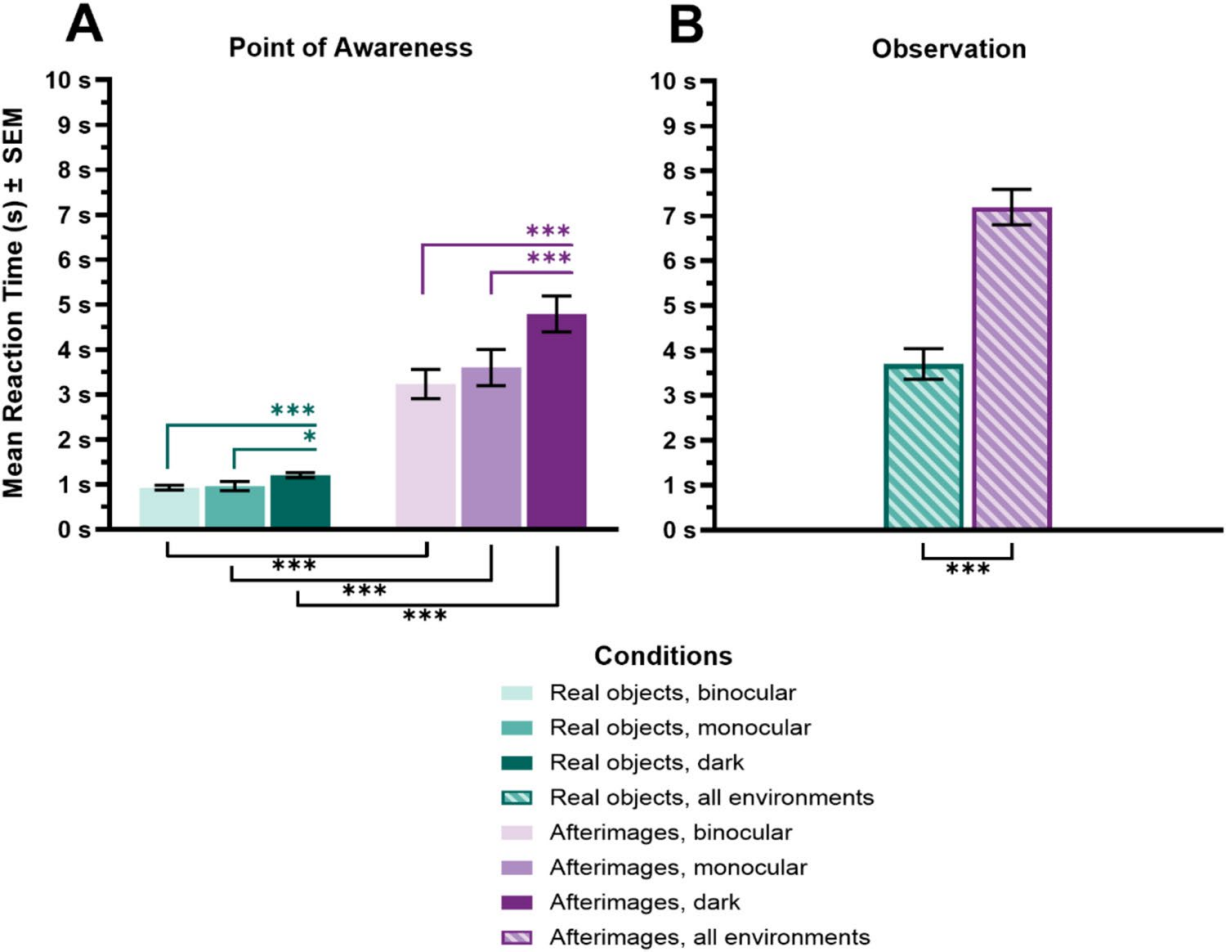
Means and standard errors (SEM) for Point of Awareness (**A**) and Observation time (**B**; in seconds; the x-axis corresponds to the matching testing conditions in the legend in all graphs). Significance indicators above the x-axis show differences between environments for the stimuli types, significance indicators below the x-axis show differences between stimuli types across environments. *p < .05, **p < .01, ***p < .001. The bars are colour coded based on condition: teal for real objects, purple for afterimages, lightest colour for binocular, medium colour for monocular, darkest colour for darkness, striped for data from all environments

ANOVA demonstrated a main effect of Visual environment (*F*_(2, 38)_ = 28.87, *p* < .001, *η*_*p*_^*2*^ = .60, *BF*_10_ = > 1000), a main effect of Stimulus type (*F*_(1, 19)_ = 63.11 *p* < .001, *η*_*p*_^*2*^ = .77, *BF*_10_ > 1000), and an interaction between the two factors (*F*_(2, 38)_ = 18.18, *p* < .001, *η*_*p*_^*2*^ = .49, *BF*_10_ = > 1000; see Fig. 7).

Pairwise comparisons (see Table 4) revealed that the interaction was driven by participants becoming aware of the real objects a few seconds faster than afterimages in all viewing conditions. However, this effect was the most pronounced in the dark, where real objects reached awareness 3.81 s faster than afterimages (monocularly 2.67 s, binocularly 2.30 s faster). Afterimages and real objects took significantly longer to reach awareness in the dark compared to both their binocular and monocular reaction times.

**Table 4:** Means and standard deviations for Point of awareness (seconds) in all conditions, and post hoc tests showing t-statistics, *p* values, Cohen’s d, and Bayesian factors

Comparison									*t*	*Pholm*	*d*	*BF10*
		*M*	*SD*				*M*	*SD*				
*Point of awareness (s)*												
Real objects	Binocular	.93	.23	–	Afterimages	Binocular	3.23	1.48	− 6.92	< .001***	− 1.88	> 1000
Real objects	Monocular	.96	.46	–	Afterimages	Monocular	3.60	1.81	− 6.59	< .001***	− 2.15	> 1000
Real objects	Dark	1.21	.25	–	Afterimages	Dark	4.79	1.79	− 8.91	< .001***	− 2.93	> 1000
Real objects	Binocular			–	Real objects	Monocular			− .35	.73	− .03	.25
Real objects	Binocular			–	Real objects	Dark			− 5.16	< .001***	− .23	> 100
Real objects	Monocular			–	Real objects	Dark			− 2.51	.04*	− .20	2.76
Afterimages	Binocular			–	Afterimages	Monocular			− 1.67	.11	− .30	.76
Afterimages	Binocular			–	Afterimages	Dark			− 6.51	< .001***	− 1.27	> 1000
Afterimages	Monocular			–	Afterimages	Dark			− 5.80	< .001***	− .97	> 1000

*p < .05, **p < .01, ***p < .001

#### Observation time (s)

The ANOVA demonstrated a main effect of stimulus type (*F*(_1, 19)_ = 22.15, *p* < .001, *η*_*p*_^*2*^ = .54, *BF*_10_ > 100), while neither the main effect of Visual environment (*F*_(2, 38)_ = .12, *p* = .89, *η*_*p*_^*2*^ = .006, *BF*_10_ = .12) nor its interaction with Stimulus type (*F*_(2, 38)_ = .94, *p* = .40, *η*_*p*_^*2*^ = .05, *BF*_10_ = .35) reached significance.

Participants spent an average of 3.49 s longer observing afterimages (*M* = 7.19, *SD* = 3.06) compared to real objects (*M* = 3.70, *SD* = 2.63) (*t* = 4.71, *p* < .001, *d* = 1.20, *BF*_10_ = > 1000; see Fig. 7).

### Colour

#### Hue (°)

**Fig. 8:**
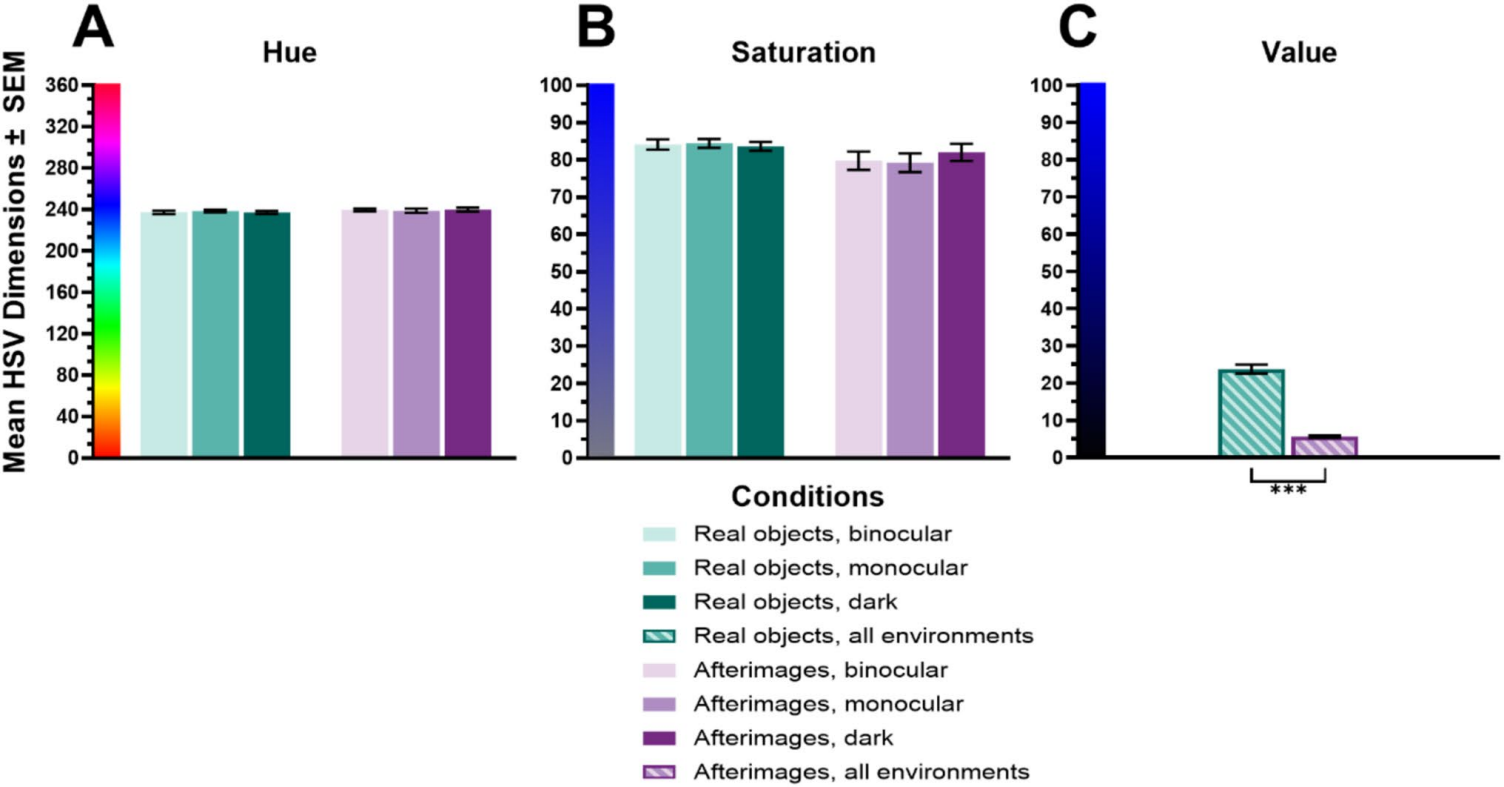
Means and standard errors (SEM) for Hue (**A**), Saturation (**B**), and Value (**C**; HSV dimensions; the x-axis corresponds to the matching testing conditions in the legend in all graphs). Significance indicators above the x-axis show differences between environments for the stimuli types, significance indicators below the x-axis show differences between stimuli types across environments. *p < .05, **p < .01, ***p < .001. The bars are colour coded based on condition: teal for real objects, purple for afterimages, lightest colour for binocular, medium colour for monocular, darkest colour for darkness, striped for data from all environments

The ANOVA demonstrated no main effect of Visual environment (*F*_(2, 38)_ = 0.03, *p* = .97, *η*_*p*_^*2*^ = .001, *BF*_10_ = .11) or Stimulus type (*F*_(1, 19)_ = 1.83, *p* = .19, *η*_*p*_^*2*^ = .09, *BF*_10_ = .68), and no interaction between the two factors (*F*_(2, 38)_ = 1.07, *p* = .35, *η*_*p*_^*2*^ = .05, *BF*_10_ = .48). All means were between 237.14° and 239.97°, which corresponds to the 240° ‘blue’ band of colour in the 360° HSV colour space (see Fig. 8).

#### Saturation (%)

The ANOVA demonstrated no main effect of Visual environment (*F*_(2, 38)_ = 0.48, *p* = .62, *η*_*p*_^*2*^ = .02, *BF*_10_ = .15), or Stimulus type (*F*_(1, 19)_ = 3.22, *p* = .09, *η*_*p*_^*2*^ = .15, *BF*_10_ = 1.21), and no interaction between the two factors (*F*_(2, 38)_ = 1.44, *p* = .25, *η*_*p*_^*2*^ = .07, *BF*_10_ = .48). All means were between 79.18 and 84.37%, indicating high purity of colour (i.e., minimal greyness) in HSV colour space (see Fig. 8).

#### Value (%)

The ANOVA demonstrated a main effect of Stimulus type (*F*_(1, 19)_ = 85.56, *p* < .001, *η*_*p*_^*2*^ = .82, *BF*_10_ > 1000), while neither the main effect of Visual environment (*F*_(2, 38)_ = 1.00, *p* = .38, *η*_*p*_^*2*^ = .05, *BF*_10_ = .19) nor its interaction with Stimulus type (*F*_(2, 38)_ = 1.55, *p* = .23, *η*_*p*_^*2*^ = .08, *BF*_10_ = .51) reached significance.

Pairwise comparisons revealed that real objects (*M* = 23.74, *SD* = 9.41) were perceived as brighter than afterimages (*M* = 5.64, *SD* = 2.70) on average (*t* = 9.25, *p* < .001, *d* = 2.59, *BF*_10_ = > 1000). Values in the range of 5% to 24% are considered as ‘dark’ in HSV colour space, however, afterimages appeared near black (see Fig. 8).

**Fig. 9:**
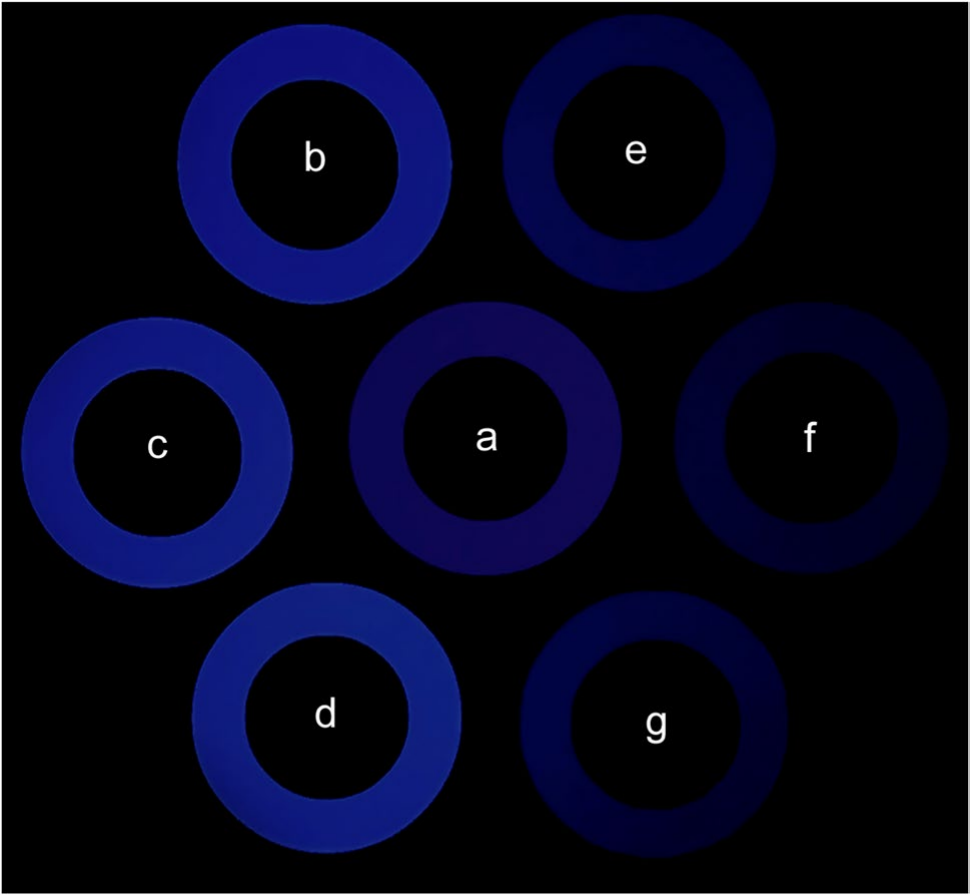
Photograph of the OLED screen displaying the averaged participant matches of the stimuli in each environment (**a**: sample of real stimuli as they appeared on screen, **b**: binocular real objects, **c**: monocular real objects, **d**: darkness real objects, **e**: binocular afterimages, **f**: monocular afterimages, **g**: darkness afterimages)

For illustrative purposes, Fig. 9 shows the averaged perceptions of real objects and afterimages for each visual environment against a sample of the real object stimuli, which all had a hue of 253°, saturation of 100%, and value of 10%. Specifically, participants’ matches were slightly bluer (i.e., less purple) and less saturated by comparison, and differed in value.

## Discussion

This study investigated whether afterimages exhibit the same degree of size constancy as real objects. We hypothesised that the differences observed in previous research may stem from the perceptual comparability of the stimuli, and that ensuring this equivalency would lead to more consistent size-distance scaling across both stimulus types. Our results showed that while both stimuli adhered to Emmert’s law under binocular conditions, afterimages deviated more in monocular and dark environments and exhibited a different relationship between their size and distance estimations. This suggests that, despite appearing perceptually similar to real objects, afterimages are processed differently by the visual system when depth cues are reduced.

The finding that size constancy mechanisms rely more heavily on depth cues for afterimages than for real objects highlights the visual system’s adaptability to stimulus-specific demands. This stimulus dependency demonstrates the flexibility of size-distance scaling mechanisms but also reveals their constraints when processing ambiguous stimuli, such as afterimages, that lack intrinsic depth cues. By clarifying these differences, this study advances our understanding of how perceptual mechanisms balance adaptability and stability across diverse visual inputs.

This distinction underscores the importance of considering stimulus-specific characteristics when interpreting size constancy mechanisms in the literature. Further, these findings imply that afterimages are inherently different as a stimulus, making them more susceptible to modulations by contextual cues. One likely explanation is that afterimages may rely on size constancy mechanisms intended for real objects, and these mechanisms may be less effective for afterimages, as their ambiguous properties deviate from the visual system’s typical expectations for real objects.

In sum, while the perceptual scaling processes cannot be considered equivalent for real objects and afterimages, this investigation highlights the value of using both stimulus types to study size constancy. Real objects provide insight into how robust perceptual mechanisms operate, while afterimages offer a way to examine the limitations of these mechanisms when depth cues are reduced. Together, these findings provide valuable insights into the flexibility of visual processing in encounters that do not fit standard visual expectations. By analysing these mechanisms across diverse stimuli, this work deepens our understanding of how the brain resolves perceptual ambiguity and maintains stability in complex environments.

### Size constancy is equivalent in typical visual conditions

Binocular, monocular, and dark visual environments were selected to represent distinct sets of depth cues, offering insight into how these cues support size constancy. Both afterimages and real objects adhered closely to Emmert’s law under binocular conditions. Size judgements were generally accurate and increased proportionally with distance. These findings align with previous work under binocular conditions, which has found the size scaling of afterimages and real objects to be largely comparable, with the caveat that afterimages exhibit some degree of instability at greater distances (Crookes 1959; Imamura and Nakamizo 2006; Lou 2007). However, we reported a modest deviation of 17% from the theoretical perfect scaling of Emmert’s law for *both* stimuli in the binocular condition, which warrants consideration of several contributing factors.

First, the absence of significant correlations between stereoacuity or visual acuity and deviations from Emmert’s law rules out the possibility that individual variability in the ability to use binocular depth cues is the primary cause. Similarly, the lack of a relationship between observation time or value and deviations from Emmert’s law indicates that differences in viewing duration and intensity of the stimuli did not meaningfully impact size or distance scaling. This is particularly relevant for afterimages, where duration and brightness can serve as indicators of their strength, making it unlikely that differences between conditions were simply due to weaker afterimages. Instead, these variations may reflect differences in participants’ capacities to perform novel perceptual tasks, a factor known to vary within the population (Baldassarre et al. 2012). This interpretation is supported by the wide range of deviations we observed across participants even under binocular conditions, from near-perfect size scaling of less than 1% deviation to those as high as 50% (see Table 1).

Our benchmarking analysis further reinforces these findings, providing a structured comparison between deviations from Emmert’s law in our study and classic findings on real objects (Holway and Boring 1941) and afterimages (Millard et al. 2020). Notably, our average binocular deviations were significantly larger than those reported by Holway and Boring, likely due to differences in participant sampling. Our study included a larger and more diverse group of naïve participants, whereas Holway and Boring’s observations were based on just five participants, including the authors themselves. This suggests that our findings may offer a more generalizable reflection of size constancy mechanisms within the broader population. In contrast, our afterimage results closely aligned with the binocular benchmark from Millard et al. (2020), reinforcing that afterimages can exhibit robust depth cue integration when both retinal and environmental cues are available.

Contextual influences may also have shaped these results. Although the experimental lighting was adequate to make the entire testing area visible, it did not mimic bright daylight conditions which might have prevented some participants from achieving higher accuracy. Although we tested a relatively large range of distances, binocular depth cues may have been less effective at the farthest distances. Vergence is thought to be ineffective at beyond 2–3 m (Tresilian et al. 1999), and the utility of stereopsis falters at around 6 m (Gregory 1973). Nonetheless, more recent investigations have shown that binocular vision can benefit from monocular information, such as perspective, and continue to enhance depth estimation accuracy up to 18 m (Allison et al. 2009).

Indeed, if distance range were the primary driver of misperceptions, we would expect to see more pronounced errors at greater distances, which was only consistent for afterimages. In fact, for real objects, size judgments tended to be overestimated when presented closer and more accurate when farther away. Such over-constancy effects can arise in controlled settings when participants are given instructions to judge ‘objective’ size (i.e., how big the object really is; Carlson 1960, 1962; Epstein 1963; Gilinsky 1955; Leibowitz and Harvey Jr., 1969; Teghtsoonian and Beckwith 1976). But since the task in this study was to judge ‘apparent’ size (i.e., how big the object appears to be) this may instead reflect our use of unfamiliar stimuli, which was a novel blue ring. Without the ability to rely on familiarity, participants who first encountered the ring at greater distances may have anchored their internal representation of its size at that point, subsequently adjusting their perception based on this initial estimate when judging it at closer distances (Sousa et al. 2013).

### Afterimages are misperceived more than real objects under reduced visual conditions

Removing use of one eye did not significantly increase errors for real objects, but had a marked effect on afterimages, which appeared undersized even at midrange distances. Variability in deviations ranged from 1% to over 100% for both stimuli, with afterimages deviating by an average of 10% more. In darkness, real objects showed significant misperceptions, yet still maintained some degree of size constancy. On average, they deviated from Emmert’s law by 40%. Although the size-distance scaling was flattened, sizes were generally judged smaller at closer distances relative to judgments at greater distances. This was not the case for afterimages which deviated on average by 80% from Emmert’s law in the dark, appearing fixed around 18–20 cm in diameter at any distance. Afterimages had a minimum deviation of 51% compared to 7% for real objects, suggesting some participants could still perform well with real objects in this condition. Overall, binocular cues relatively contributed just 3% to size scaling effect for real objects, and did not uniquely contribute at any distance. They contributed 8% for afterimages and provided distinctive improvements at distances beyond 4 m. Monocular cues also had relatively minimal impact on real objects (16%) but accounted for 65% of afterimage scaling. The information left over in darkness accounted 30% of the effect for afterimages, and higher weight of 75% of the effect for real objects.

From this, we can conclude that binocular and monocular cues play a critical role in stabilising size perception for afterimages, whereas real objects can still be processed with some level of constancy using lower-quality depth information. The stark contrast between perceptually similar afterimages and real objects in reduced environments highlights that afterimages must rely on different (or less robust) mechanisms, compared to real objects, and that the visual system is less able to integrate the various sources of depth cues with afterimages. We have previously reported on the importance of binocular cues for maintaining size constancy in afterimages (Millard et al. 2020) and provide more evidence for this here. These findings are also consistent with early comparisons, where real stimuli maintained size constancy more reliably than afterimages under monocular reduction (Edwards 1953; Furedy and Stanley 1970, 1971; Hastorf and Kennedy 1957).

It is worth noting that our monocular condition did not restrict the pupil, allowing accommodation to remain effective. Some studies, such as Irwin’s (1969) comparison and Holway and Boring’s (1941) classic study with real stimuli (see also Sperandio et al. 2009), included the use of pinholes or tunnel viewing to eliminate accommodation by limiting the light entering the eye, thereby reducing the need for the lens to adjust for focus. In such conditions, they report that size perception more closely followed retinal image size. Our more naturalistic monocular viewing results did not exhibit full regression to the visual angle and closely aligned with Holway and Boring’s (1941) monocular condition that did not use a pinhole. This further supports the reliability of monocular depth cues in maintaining size constancy for real objects across different experimental designs. Despite significant misperceptions in our dark condition, adherence to Emmert’s law was notably stronger than in Holway and Boring’s (1941) darkness benchmark, where the use of a pinhole and reduction tunnel eliminated oculomotor cues.

This likely reflects the contribution of oculomotor adjustments, which were preserved in our more naturalistic setup. It has been demonstrated that the presence of a fixation point in an otherwise completely dark environment provided sufficient reference for participants to adjust their vergence angle and influence perceived size (Sperandio et al. 2013). In the current experiment, distances beyond the effective range of vergence were tested, and there was no fixation points provided during the observation phase to allow for the environment to be as dark as possible. However, the real stimuli were likely able to act as a fixational anchor on their own, which would allow the eyes to verge on their location to some degree.

In contrast, afterimages in the dark exhibited a greater breakdown of size constancy and lacked a tangible presence for oculomotor adjustments. Yet, their perception also did not fully align with retinal image size (i.e., the 4 cm inducer). With both eyes unrestricted in the darkness condition, residual oculomotor cues may have influenced perception. This is further supported by our benchmarking analysis, which compared these findings to Millard et al. (2020), where an eyes-closed condition was used to create total darkness in a similar afterimage paradigm. The reduced deviations from Emmert’s law in our study suggest that even minimal residual depth cues, absent in the prior study, contributed to greater perceptual stability. However, the smaller monocular deviations relative to the benchmark may also reflect methodological differences in this study, such as the use of an adjustable matching tool and an extended distance range. These refinements likely facilitated more precise size estimations compared to the categorical matching approach and shorter distance range in our prior work.

However, this does not negate that even with the eyes closed, we found that afterimages did not reflect retinal size even when projected onto the eyelids. Rather, size varied across individuals. We attributed this to specific distance tendency, where vergence and accommodation assume an intermediate position in total darkness (Gogel 1965; Gogel and Tietz 1977; Owens 1984). In a demonstration of this, it has been found that participants cannot voluntarily alter afterimage size in the complete absence of visual information through oculomotor control (Suzuki 1986).

Taken together, participants in this study may have been able to accommodate and verge on real objects in the dark due to having naturalistic, unrestricted viewing, whereas afterimages likely caused the eyes to lock into a resting state. For real objects to be perceived clearly in complete darkness, accommodation and vergence must still occur to prevent blurriness and double vision. The presence of a physical stimulus provides the necessary input to guide these oculomotor adjustments, ensuring a stable retinal image. In contrast, afterimages lack an external reference, preventing effective accommodation and vergence, which may explain their greater misperception in darkness. This distinction could explain the stunted size-distance scaling for real objects, the greater deviations from Emmert’s law for afterimages, and the lack of retinal size perception for either stimulus type in darkness. This interpretation is further supported by our data, with the dark condition accounting for 45% more of the size scaling effect for real objects compared to afterimages.

One alternative consideration is that the slight preservation of size-distance scaling in some participants in darkness may be due to the faint glow from the real stimuli on the OLED screen. Importantly, this refers not to the OLED screen itself, but the minimal luminance emitted by the stimuli. Though quite dark at 10% value, we anticipated this as a potential factor and measured luminance levels at all distances (average readings from near distances where the stimuli were physically smallest were 0.141 cd/m^2^, further distances where the stimuli were physically largest were 0.160 cd/m^2^). Additionally, we assessed ambient luminance in the testing space surrounding the stimuli at each distance (walls, ceiling, and floor) during pilot testing, which remained consistently dark (average reading of 0.00275 cd/m^2^). While these measurements suggest minimal ambient interference, we acknowledge the possibility that participants with heightened light sensitivity could have perceived subtle linear perspective cues.

This idea is supported by the substantial variability in deviations observed in darkness for real objects (ranging from 7 to 88%)—if a strong cue were present, one would expect more consistent effects across participants. Additionally, a recent experiment has shown that even minimal linear perspective, such as low-light LED strips lining a dark tunnel, can be sufficient for accurate size judgments (Norman et al. 2022). Although no direct relationship was found between perceived brightness and deviation from Emmert’s law, the luminous nature of the stimuli may have influenced perception in ways that are difficult to quantify. It remains possible that more light-sensitive participants could have used this faint cue.

Our findings could also be interpreted within conceptual frameworks that posit an interplay between “bottom-up” and “top-down” mechanisms (e.g., Rauss and Pourtois 2013; Teufel and Nanay 2017). Under such frameworks, our results suggest that afterimages rely predominately on bottom-up processes, automatically scaling their perceived size based on available environmental depth cues. The strong adherence to Emmert’s law observed under binocular viewing, and the pronounced breakdown in size-distance scaling when depth cues were reduced in monocular or darkness conditions, underscores the dependence of externally derived depth information. In contrast, real stimuli, despite being unfamiliar objects, exhibited greater perceptual stability across reduced visual conditions, suggesting that top-down influences, such as those derived from perceptual hypotheses, partially compensate when bottom-up sensory cues become impoverished. Thus, the differential scaling behaviour we observed between real stimuli and afterimages across visual environments could reflect an interplay between sensory (bottom-up) and interpretive (top-down) processes in human size perception.

### Perception of size and distance were partially uncoupled

Distance perception exhibited similarities to size perception. Like size, distance perception was equivalent between real objects and afterimages under binocular conditions, adhering to Emmert’s law. However, this coupling broke down under reduced conditions, where afterimages were more vulnerable to misperceptions. Distance judgments of real objects remained similar in binocular and monocular conditions but worsened in darkness. For real objects, the cue contributions for both size and distance scaling were similar, with binocular cues being negligible, monocular cues contributing modestly, and most of the scaling driven by information available in the dark. In contrast, monocular cues accounted for almost all the effect (90%) for afterimages, with binocular and dark cues contributing less (less than 1% and 8%, respectively).

Importantly, the nature of the misperceptions driving the deviation from Emmert’s law differed across conditions. In binocular conditions, there was better adherence to Emmert’s law for both afterimages and real objects when estimating distance across the full range. Under monocular conditions, distance was consistently overestimated, while size was often underestimated at farther distances. In darkness, distance judgments of real objects were more prone to underestimation, while afterimages were perceived at a midrange distance (approximately 4 m), which did not align with the smaller sizes they were judged to have (corresponding to about 2.5 m).

Correlation analyses further support distinct processing for size and distance. Distance misperceptions were more uniform across stimulus types and environments, indicating greater precision in distance discrimination than size discrimination. This aligns with observations made by Kaufman and colleagues (2006). In their study they proposed that the greater noise in size judgments may reflect the added uncertainty of integrating angular size and distance information. For real objects, the proportional relationship between size and distance only held in reduced environments, suggesting that in rich visual settings, size perception may rely on factors beyond distance cues. For afterimages, size and distance shared variance in binocular and monocular conditions but not in darkness, implying a common scaling mechanism that breaks down with limited depth information.

Some of the disparity between size and distance perception could stem from the methods used to capture each measure. Distance was measured using a verbal magnitude estimate, which is more intuitive but prone to cognitive bias (Dong et al. 2023). In contrast, size was assessed more concretely, requiring participants to adjust a reference stimulus, a task that likely involved more complex mental processing. This may have made it harder to integrate size and distance as would occur naturally in everyday viewing. However, alternative distance measures, like blind walking, were not suitable due to the experimental setup, and there is a body of evidence showing similar independence of size and distance perception (e.g., Brenner and van Damme 1999; Gruber 1954; Haber and Levin 2001; Kim et al. 2016). Thus, the differences in measurement likely do not fully account for our results, as we would expect consistent discrepancies across all conditions if they did. Even with some uncoupling due to measurement differences, size and distance judgments diverged meaningfully depending on stimulus type and available depth cues.

How then, do theoretical frameworks like Emmert’s law and SDIH hold when they assume a one-to-one relationship between size and distance? It has been argued that these frameworks are flawed and do not apply to all circumstances (see Ross 2003 for review). Their reliance on monocular geometry may not fully account for binocular vision (see Kim 2017, 2018 for recent critiques and suggested revisions). Though this may be the case, it does not explain why size perception still follows Emmert’s law in rich visual environments. One possibility is that participants are asked to make conscious judgments about a process that is typically automatic. Explicit judgments of apparent size or distance may conflict with the underlying, unconscious processing of depth.

This suggests two levels of distance processing: an unconscious estimate influencing size perception via mechanisms like Emmert’s law, and a conscious distance report shaped by confidence and available depth cues. This idea is supported by theories from literature on the Moon Illusion, which propose that size perception is driven by automatic distance registration, while conscious judgments may not always align with these processes (Kaufman and Rock 1962; Ross and Plug 2002). Further evidence for automatic distance processing comes from the dual visual stream theory (Goodale and Milner 1992), which posits that the visual system has dual representations of size: one for conscious perception of objects and another for unconscious, action-related processing needed for interacting with objects.

### On achieving comparability between afterimages and real objects

The temporal profiles of the stimuli were distinct, which was expected given the differences in their nature. Afterimages, being transient, took longer to manifest as evidenced by our results, whereas real objects, with their stable presence, were perceived more quickly. Notably, both afterimages and real objects showed delayed awareness in the darkness condition, likely due to the absence of the many contextual cues that aid perception in other environments. In darkness, the visual system struggles to localise stimuli due to the diminished availability of environmental information. Interestingly, this delay occurred despite the luminescent real stimuli and the sudden onset of darkness. This darkness condition used in our experiment is not representative of typical nighttime vision as illumination was rapidly extinguished for each trial, which did not allow participants to adjust to the low level of light. Future research could explore whether such delays persist after dark adaptation, potentially informing driver awareness of fluorescent pedestrian garments in low-light conditions.

Afterimages exhibited greater delays in awareness, reinforcing their stronger reliance on binocular and monocular cues compared to real objects. In our previous work (Millard et al. 2020), afterimages lasted longer in the eyes-closed condition, but here, darkness alone did not prolong their visibility. Both afterimages and real objects stabilized after initial perception, suggesting that looking into complete darkness differs fundamentally from eyes-closed darkness, or that the high-contrast afterimage inducer led to more robust afterimages.

Aside from timing differences, the visual environments did not significantly impact other measures of perceptual similarity. The retinal image size, hue, and saturation of both stimulus types were almost certainly identical, with our study being the first to systematically match comprehensive perceptual profiles of externally and internally generated stimuli. By focusing on objective colour dimensions rather than subjective reports, we ensured that the stimuli were perceptually equivalent across several dimensions. However, one notable exception was value, where neither stimulus was perceived in line with the target colour.

Real objects were perceived as more than twice as bright as they were, while afterimages appeared around half as bright as anticipated. The fuzziness and tendency of afterimages to blend into their surroundings likely played a key role in this discrepancy. We theorize that afterimages may have ‘absorbed’ some of the black OLED screen background, making them appear darker. Supporting this, Powell et al. (2012) found that adding a sharp luminance edge around an afterimage increases its contrast from the background, whereas this edge had little impact on real objects. In other words, stabilising the fuzzy edges of afterimages makes their visual signal behave more like real objects, which already have well-defined boundaries. For real objects, this high visibility of their edges may have contributed to their increased brightness perception. The OLED screen’s black background, created by turning off all diodes except those for the stimuli, resulted in a stark contrast with the luminance of the ring. Similar arrangements have been shown to cause the visual system to infer differences in illumination, leading to altered brightness perception (Maertens et al. 2015; Schirillo and Shevell 2002).

### Implications for neural processing of afterimage signals

Our findings further support the idea that afterimages have a cortical locus of adaptation (see Shimojo et al. 2001; Tsuchiya and Koch 2005; Sperandio et al. 2012a, b; see also Sperandio et al. 2012a, b for evidence in the context of size constancy). Here, if afterimages were primarily driven by retinal signals, they would be perceived similarly across environments, as the retinal image size remains constant regardless of external depth cues. In that scenario, retinal aftereffects would dominate, with less environmental influence on size and distance perception. However, the variability in afterimage perception across conditions, especially their reliance on monocular cues and the significant breakdown of size constancy in darkness, suggests that cortical processes are involved.

Our results also lend credence to the signal ambiguity theory (see Powell et al. 2012, 2015, 2016 for current perspectives, see also Brindley 1962 and Lupyan 2015 for relevant demonstrations, though not directly discussed as 'signal ambiguity theory'), which posits that afterimages are ambiguous signals because they lack a stable external reference. As a result, the brain relies more heavily on contextual information (such as prior knowledge, surrounding cues, and internal predictions) to interpret these signals. This makes afterimages more susceptible to being influenced by external factors, affecting whether they are perceived as stable or interpreted like real objects.

The absorption of the black background by afterimages, and their failure to exhibit robust size-distance scaling in reduced cue environments, highlights that afterimages were processed differently than real objects and were more vulnerable to contextual influences. This suggests that size-distance scaling mechanisms have evolved to process real-world stimuli, and when afterimages resemble real stimuli, they can exhibit size constancy. However, when afterimages present atypical characteristics (such as jittery movements, fading, or blurred edges) the brain interprets them as ‘not real’, leading to their suppression. As a result, the scaling mechanisms either fail to apply or function differently than they do for real objects.

## Conclusions

We have presented evidence that afterimages and real objects, though perceptually comparable under certain viewing conditions, are processed differently by the visual system, particularly when depth cues are reduced. The comparisons from the benchmarking model serve to reinforce the critical role of depth cue availability in determining the success of size constancy mechanisms for both real objects and afterimages. While both can be used to examine size constancy, our findings suggest that afterimages, unlike real objects, rely more heavily on contextual information and may represent a residual or misappropriated effect of the size constancy system designed for external stimuli. When afterimages become part of conscious experience, they seem to activate mechanisms typically reserved for real objects, yet their instability under reduced visual cues highlights the limitations of these processes. However, this instability also underscores the unique value of afterimages as a tool for probing the boundaries of visual perception, offering insights into how the brain processes ambiguous or incomplete visual information.

## Data Availability

The data supporting the findings of this study are available on the Open Science Framework (OSF) at https://osf.io/qabgz/?view_only=e30f97cbf83a44858ff076eee998fce6.
